# Impact of Holder Pasteurization and Preanalytical Handling Techniques on Fat Concentration in Donor Human Milk: A Scoping Review

**DOI:** 10.1016/j.advnut.2024.100229

**Published:** 2024-05-30

**Authors:** Autumn Davis, Maryanne T Perrin

**Affiliations:** Department of Nutrition, University of North Carolina at Greensboro, Greensboro, NC, United States

**Keywords:** Holder pasteurization, donor milk, human milk, mixing, lipids

## Abstract

**Background:**

Donor human milk (DHM) is an essential source of nutrition among high-risk infants (e.g., premature and low-birth weight). Holder pasteurization, a common step in DHM processing, is known to partially alter the composition of DHM; however, the impact on fat composition is historically inconsistent.

**Objectives:**

This scoping review aimed to broadly review the literature on the impact of Holder pasteurization on the fat content in DHM, with a focus on preanalytical sample mixing.

**Methods:**

A systematic search of original, peer-reviewed research articles was conducted on 11 July, 2022. Articles were included if they compared matched raw (control) and Holder-pasteurized human milk samples and measured total lipids, cholesterol, and individual classes of fatty acids. Article review and selection was conducted by 2 independent reviewers.

**Results:**

The search yielded 26 original, peer-reviewed research articles published between 1978 and 2022. Overall methodology varied considerably between studies. When study methods described any mixing for collecting raw milk, 1 (17%) of the 6 of studies reported a small change in total fat concentration following pasteurization (<5%). Alternatively, among studies that did not describe methods for mixing raw milk to ensure a representative sample, 10 (56%) of the 18 reported a significant change (≥± 5%) in total fat concentration, with changes ranging from −28.6% to +19.4%.

**Conclusions:**

This review suggests that inconsistent findings regarding the impact of Holder pasteurization on fat may be related to study methodologies, particularly preanalytical sample mixing. More research considering the role of preanalytical handling procedures and methodologies is necessary to help clarify the impact of Holder pasteurization on human milk composition.

**Statement of Significance**

To our knowledge, this review was the first to explore how sample handling techniques influence findings related to lipid retention in human milk after Holder pasteurization.

## Introduction

Human milk is currently considered the gold-standard source of nutrition for infants, and its use is recommended by the American Academy of Pediatrics to be the reference standard to which all other infant feeding practices are compared [1]. Human milk is uniquely suited for providing the newborn with the nutrients, bioactive, and immune-boosting factors necessary for growth and development [1,2]. When a mother milk is not available, donor human milk (DHM), appropriately fortified, is considered the next best option, especially among premature or low-birth weight babies weighing <1500 g [3].

In the United States, DHM is processed by a milk bank before it is delivered to neonatal intensive care units and clinics. Most milk banks adhere to the guidelines of the Human Milk Banking Association of North America to ensure the safe collection and distribution of DHM. Processes include screening and approving donors; receiving and storing raw milk; thawing, pooling, mixing and bottling; pasteurizing; and testing DHM batches for bacterial contamination [4].

Although milk bank processing effectively rids DHM of bacterial and viral contaminants, there is concern that processing alters the composition of DHM and that this coincides with the early research reports of slower growth rates among preterm infants fed pasteurized DHM [5]. Evidence supports that Holder pasteurization results in the loss of some beneficial DHM components, such as enzymes, certain immunoglobulins, amino acids, and vitamins [6]; however, for other components, the effect is still unclear. There is a notable lack of agreement among studies reporting the impact of Holder pasteurization on DHM fat. For instance, reported reductions in fat concentration postpasteurization have varied from as little as “no loss” to as much as 25% [7,8]. These discrepancies are concerning because dietary fat plays a critical role in providing the energy and functional structures necessary for supporting optimal growth outcomes among high-risk infants [9].

Although evidence regarding the impact of Holder pasteurization on fat content is conflicting, it is necessary to consider that a change in DHM composition may be relative to other stages of milk processing**.** For instance, the 2018 Pediatric Nutrition Practice Group’s publication *Infant Pediatric Feedings* acknowledges that fat changes may be influenced by differences in DHM sample containers, inadequate thawing, and poor mixing, among other factors [10]. Additionally, a recent publication by Friend and Perrin [11] explored the impact of mixing and bottling raw DHM and found significant differences in fat distribution based on the mixing method and storage time of pooled milk.

Without consensus as to the impact of Holder pasteurization on DHM fat, the primary aim of this scoping review was to summarize study findings and methodologies regarding the observed impact of Holder pasteurization on the retention of fat in DHM. Additionally, considering the evidence suggesting that mixing methodologies may play a significant role in the outcomes of DHM fat distribution, the potential relationship between mixing methodologies and pasteurization outcomes will also be explored.

## Methods

We conducted a scoping review informed by the work of Arksey and O’Malley [12]. A systematic search was conducted to identify original research articles published through 11 July, 2022, that reported quantitative data on the lipid content of raw and Holder-pasteurized DHM. The review process was conducted according to the PRISMA checklist. Electronic searches of CINHAL, PubMed, and SCOPUS were carried out using the following search queries: [Fat OR lipid OR macronutrient) and (Holder OR pasteuriz∗) and (“donor milk” or “donor human milk” or “human milk”)]. Search limits included peer-reviewed publications and published in English. Reviews and abstract briefs were excluded from the analysis.

Following the initial search, all database returns were reviewed and deduplicated (ARD) to only include original publications. The abstract for each original publication was then screened by 2 researchers (ARD and MTP). There was no restriction on publication dates; abstracts were excluded if they did not mention DHM, did not conduct a pre–post pasteurization analysis of DHM, or did not consider the fat composition of DHM. Abstracts meeting the criteria were advanced for full article review by 2 researchers (ARD and MTP).

Studies were excluded if the research design did not specify Holder pasteurization (62.5–63 °C for 30 min); if the comparison of DHM samples before and after Holder pasteurization was not matched; if DHM fat content data were not reported as concentration for both raw and Holder pasteurization DHM (data reported as proportion of total fat was not included); or if treatment of raw and pasteurized samples were incongruent (e.g., pasteurized samples underwent additional freeze–thaw cycle). Further, reported data must have been preabsorption (fecal and gastric digestive data were not included); data reported on total lipids, cholesterol, and individual classes of fatty acids were considered eligible.

Studies that passed the full-text review were abstracted (ARD and MTP) for the following information: funding source; milk characteristics: (sample size, number of donors or pools, term/preterm delivery, and average lactation stage); characteristics of pasteurization (pasteurization equipment and volume); analyte data (fat analytes studied and analysis methods); pool mixing details of raw and Holder-pasteurized samples; and descriptive statistics. Additionally, references of included studies were hand searched to identify other studies for consideration. When the same underlying milk samples informed more than 1 study, results were only included if they reflected unique analytes in the milk.

The main outcome variable was the percent change in milk fat concentration, because fat is highly variable in human milk, thus percent change would allow comparison across a range of fat values. Percent change in fat was computed using the descriptive statistics that were abstracted for raw and matched Holder-pasteurized samples (mean unless otherwise noted). Data were organized based on the detail provided in a study’s methodology regarding the mixing of a representative milk sample.

**FIGURE 1:**
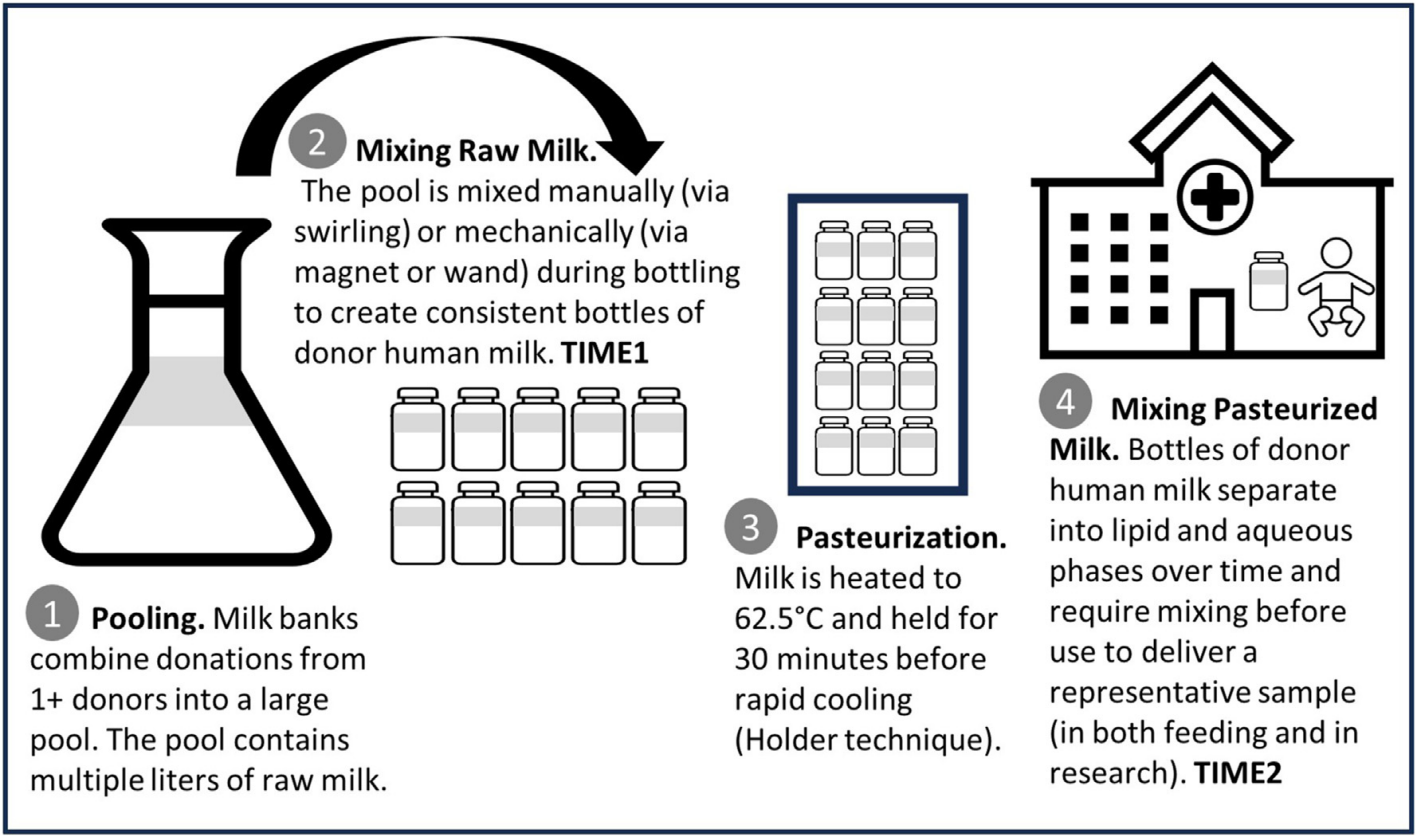
Overview of donor milk production and key time points for mixing to ensure creation of a representative sample.

Although bovine milk in the United States is homogenized to create an emulsion of the lipid and aqueous fractions that does not separate over time [13], currently, nonprofit milk banks do not homogenize human milk in order to protect its many bioactive molecules; thus, DHM separates over time. Mixing refers to the process of agitating DHM so that the fat and aqueous layers are adequately incorporated, with the goal of ensuring “representative” milk when subdividing (e.g., bottling DHM at the milk bank; creating DHM feeding syringes in the hospital; or sampling DHM for research). For the purpose of this review, the 2 following time points were determined to be critical for obtaining a representative DHM sample: *1*) just before aliquoting the raw DHM samples and bottling the pooled milk and *2*) following pasteurization when drawing a sample for fat analysis. Figure 1 provides an overview of these points within the framework of the production and use of DHM.

Data were coded independently by 2 reviewers (ARD and MTP) according to the degree of mixing and any discrepancies were resolved by discussion. A “high” degree of mixing was assigned if the study used a mechanical mixing instrument (e.g., ultrasonic homogenizer and vortexer) and disclosed the temperature of the milk pool/sample(s) at the time of mixing. Mixing techniques were classified as “moderate” if temperature information was provided, and mixing techniques were nonmechanical (e.g., manually mixed via inversion or pour-down method). A “low” degree of mixing was assigned if mixing techniques were nonspecific (e.g., “the pools were mixed”), and there was no presence of temperature information. When no mixing information was provided, mixing was classified as “no information.”

## Results

### Study selection

**FIGURE 2:**
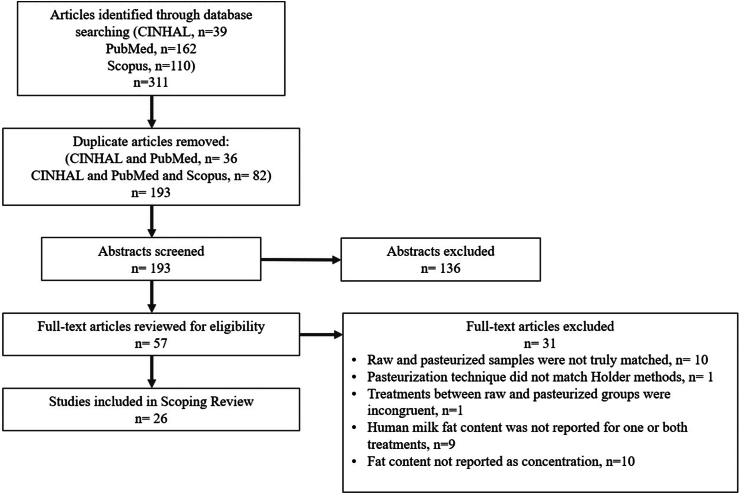
Flowchart summarizing article inclusion and exclusion process.

Initial database searches returned 162 articles from PubMed, 110 articles from Scopus, and 39 articles from CINHAL. Following the removal of duplicate articles and abstract reviews, 57 articles remained for full-text review. Thirty-one articles were excluded after full-text review, leaving 26 articles included in the scoping review (Figure 2) [7,8,[14], [15], [16], [17], [18], [19], [20], [21], [22], [23], [24], [25], [26], [27], [28], [29], [30], [31], [32], [33], [34], [35], [36], [37]]. The hand search of references did not identify additional studies that met the inclusion criteria.

### Study and sample characteristics

Articles were published between 1978 and 2022 (Table 1) with most studies (21/26, 81%) conducted in the last 15 y, highlighting the growing interest in DHM as a feeding therapy. When the number of individual donors was reported, it ranged from 2 to 191. When the number of samples or pools was reported, it ranged from 1 to 460. Fifteen of the studies (58%) reported the use of only mature DHM (samples collected >15 d after birth); 2 studies analyzed only transitional DHM (collected 5–15 d postpartum), and 2 studies included DHM samples from multiple lactation stages (colostrum, transitional, and mature).

**TABLE 1:** Summary of study characteristics, DHM samples/pools, and pasteurization qualities

Author, year	Funder	Characteristics of milk (No. of donors/samples; gestation; lactation stage)	Pasteurizing equipment	Pasteurizing volume	Fat analytes studied
Williamson et al., 1978 [14]	No funding information provided	7 pools; NA; mature milk	—	—	Total fat
Friend et al., 1983 [15]	NIH/NICHD	4 pools (8–12 samples per); NA; mature milk	—	—	Total Fat
Lepri et al., 1997 [16]	No funding information provided	Donor *n* = 16; average gestation: 38.6 wk; NA	—	—	Total fat
Fidler et al., 2001 [17]	Deutsche Forschungsgemeinschaft and Deutscher Akademischer Austauch Dienct, Bonn, Germany	Donor *n* = 12; 66% preterm; transitional milk (median value)	Thermoblock	2 mL	Total fat
Góes et al., 2002 [18]	CNPq, FAPERJ, CAPES, FINEP, and FUJB	15 single donor pools; NA; mature milk	—	—	Total fat
Ley et al., 2011 [19]	Canadian Diabetes Association; Canadian Foundation for Dietetic Research; Canadian Institutes of Health Research	Donor *n* = 34 (17 pools); NA; mature milk	Breast milk pasteurizer (T30/USA Sterifeed)	—	Total fat
Vieira et al., 2011 [7]	CNPq (translate to English) Brazilian National Council for Scientific and Technological	57 pools; NA; NA	—	74 mL	Total fat
García-Lara et al., 2013 [20]	Spanish Health Research Funding and Spanish Collaborative Research Network	Donor *n* = 28 (34 samples); average gestation: 36.6 wk; mature milk	Thermostatic Bath—continuously stirred	60 mL	Total fat
Kotrri et al., 2016 [21]	Canadian Institute of Health Research	50 samples	—	50 mL	Total fat
Adhisivam et al., 2019 [8]	No funding information provided	Donor *n* = 90 (30 pools); average gestation: 36.5 wk; transitional milk	Shaker-water bath	—	Total fat
Lima et al., 2017 [22]	Funded by North Carolina State University	Donor *n* = 60 (1 pool); NA; NA	Ace intermed special feed pasteurizer	88.7 mL	Total fat
Piemontese et al., 2019 [23]	No financial support was provided for research	Donor *n* = 191 (460 samples); 90.9% full term; mature milk	S90 Eco sterifeed pasteurizer	10 mL	Total fat
Castro et al., 2019 [24]	Canadian Institutes for Health Research	Donor *n* = 8; full term; mature milk	—	—	Total fat
Pitino et al., 2019 [25]	Canadian Institutes of Health Research	Donor *n* = 8 (10 pools); NA; NA	Sterifeed tabletop pasteurizer	125 mL	SFA, MUFA, PUFA
Pitino et al., 2019 [26]	Canadian Institutes of Health Research	Donor *n* = 17; NA; NA	Sterifeed Tabletop pasteurizer	125 mL	Total fat
Chang et al., 2020 [27]	No financial support was provided for research	Donor *n* = 100 (1 pool); NA; mature milk	—	—	Total fat
Vincent et al., 2020 [28]	SFMP (French Society of Perinatal Medicine)	Donor *n* = 2; full term; mature milk	—	—	Total fat, Total fatty acids (esterified and non-esterified)
Jorge dos Santos et al., 2021 [29]	Coordenaçao de Aperfeiçoamento de Pessoal de Nível Superior - Brasil (CAPES), Conselho Nacional de Desenvolvimento Científico e Tecnologico ´ (CNPq), and Fundaçao Arauc´ aria	Donor *n* = 22–23 (68 samples); NA; colostrum sample *n* = 22, transitional and mature milk sample *n* = 23 each	Water bath, with stirring	50 mL	Total fat
Escuder-Vieco et al., 2021 [30]	Spanish Research Projects in Health, Hero Institute for Infant Nutrition, RETICS “Maternal and Child Health and Development Network”	Donor *n* = 48 (10 pools); 80% full term; mature milk	Shaking water bath: Jeio Tech BS-21; Lab Companion	120 mL	Total fat
Lamb et al., 2021 [31]	No financial support was provided for research	Donor *n* = 27 (63 pools); 79% full term; 97% mature milk	Water bath	—	Total fat
Quitadamo et al., 2021 [32]	No information provided	Donor *n* = 87 (100 pools); average gestation = 37.6 wk; NA	—	—	Total fat
Caballero Martín et al., 2022 [33]	No financial support was provided for research	Sample *n* = 8; NA; mature milk	Beldico PA 45 pasteurizer (water-free)	mixed volumes: 40–120 mL	Total fat
Pitino et al., 2022 [34]	Canadian Institutes of Health Research, Hospital for Sick Children institute	Sample *n* = 10; NA; mature milk	Shaker-water bath	9 mL	Total fat
Jorge dos Santos et al., 2022 [35]	Coordenaçao de Aperfeiçoa mento de Pessoal de Nível Superior—Brasil (CAPES), Conselho Nacional de Desenvolvimento Científico e Tecnologico (CNPq)	Donor *n* = 3 (3 pools per lactation stage); NA; samples from colostrum, transition and mature milk stages	Stirring water bath	60 mL	Total fat
Martysiak-Żurowaska et al., 2022 [36]	National Science Center Poland	Donor *n* = 25; full term; mature milk	Water bath	50 mL	Total fat
Ten-Doménech et al., 2022 [37]	Instituto de Salud Carlos III, Spain; the Ministry of Science and Innovation, Spain, the European Union’s Horizon 2020 Research and Innovation Programme	Sample *n* = 12; average gestation: 40 wk; mature milk	—	—	Specific fatty acids, SFA, LCFA, MUFA, PUFA

Abbreviations: LCFA, long-chain fatty acid; NA, not available.

Holder pasteurization equipment and pasteurization volumes varied and are detailed in Table 1. Only 15 studies described the pasteurization equipment used, of which, about half (*n* = 8) reported using a water bath, whereas others (*n* = 7) used a specialized pasteurizer or other method. Pasteurization volume was described in over half of the studies (15/26, 58%), with reported volumes ranging from 9 to 125 mL. Total fat was the most popular analyte examined (24/26, 92% of studies) with limited studies on individual fatty acids and classes of fatty acids.

### Results by mixing information

Of the 26 studies reviewed, 10 studies (38%) provided some information on mixing protocols [17,20,22,23,25,26,31,32,34,37]. Table 2 summarizes these studies by analyte and the degree and quality of mixing at the following 2 key time points: *1*) representative raw samples and *2*) representative Holder-pasteurized samples. The reported time points, and quality of mixing varied among the studies. Two studies reported mixing protocols for both time points, whereas 6 of the 10 studies reported mixing only for the raw sample, and 2 of the 10 studies reported mixing only for the Holder-pasteurized sample. Mixing of the raw samples included high-quality mixing (2/8), moderate-quality mixing (3/8), and low-quality mixing (3/8). Mixing of the Holder-pasteurized samples include high-quality mixing (3/4) and low-quality mixing (1/4). Of the 6 studies that assessed total fat and described mixing at the first time point to achieve a representative raw sample [17,20,22,26,31,34], only 1 (17%) reported a small but significant loss (4.1%) in total fat after pasteurization [20]. In contrast, 2 (100%) of the 2 of studies that described mixing only the Holder-pasteurized samples found significant losses in total fat concentrations by 3.4% and 19.4% [23,32]. Results among the 2 studies that analyzed fatty acid concentrations—including total fatty acids, MUFAs), PUFAs, SFAs), and long-chain fatty acids—and described mixing techniques (Table 2) were inconsistent [25,37]. Both studies reported mixing at the first time point; however, 1 described low-quality mixing and reported significant losses ranging from 12.5% to 26.5% [37], whereas the other described moderate-quality mixing and found no significant changes [25]. Only 1 study with low-quality mixing quantified changes in individual fatty acids and results ranged from a loss of 38% to a gain of 17% [37].

**TABLE 2:** Summary by analyte and degree of mixing for matched raw and Holder-pasteurized donor human milk among studies that reported mixing information in the methods

Analyte	Author	Analysis method	Pool mixing and temperature: time 1^1^ (technique)	Pool mixing and temperature: time 2^2^ (technique)	Raw value	HoP value	% Change	*P*
Total fat	García-Lara et al. [20]	Mid-infrared human milk analyzer	HIGH (40°C; homogenized with ultrasound homogenizer)	HIGH (40°C; homogenized with ultrasound homogenizer)	Mean (95% CI): 4.9 (4.2, 5.6) g/dL	Mean diff (95% CI): −0.2 (−0.3, −0.04) g/dL	−4.1%	Not reported
	Pitino et al. [34]	Mid-infrared human milk analyzer	HIGH (4°C; inverted 10 times and vortexed intermittently for 1 min)	—	Median (1stQ, 3rdQ): 3.3 (2.8, 3.8) g/dL	3.1 (2.8, 3.8) g/dL	Not significant	—
	Pitino et al. [26]	Mid-infrared human milk analyzer	MODERATE (37°C; agitated and gently inverted to ensure homogeneity)	—	Mean ± SD: 31 ± 8 g/L	31 ± 8 g/L	Not significant	—
	Lima et al. [22]	NMR-based Smart Trac analyzer	MODERATE (≤4°C; gently swirled in Erlenmyer flasks for 3 sec after manually mixing pool with “pour down” method)	—	Mean (SD): 3.9% ± 0.04% fat	3.9% ± 0.1% fat	Not significant	—
	Fidler et al. [17]	Gravimetric (Roese-Gottlieb)	LOW (unspecified “mixed”)	LOW (38°C; unspecified “mixed”)	Mean ± SE: 3.1 ± 0.4 g/dL	3.1 ± 0.4 g/dL	Not significant	—
	Lamb et al. [31]	Mid-infrared human milk analyzer	LOW (stirred, aliquoted)	—	Mean ± SD: 3.5 (SD = 1.0) g/100 mL	3.4 ± 1.0 g/100 mL	Not significant	—
	Piemontese et al. [23]	Mid-infrared human milk analyzer	—	HIGH (35–40°C; ultrasonic homogenizer (sonicator: 1.5 s/mL)	Mean (SD): 2.9 ± 0.9 (g/100 mL)	2.8 ± 0.8 g/100 mL	−3.4%	<0.0001
	Quitadamo et al. [32]	Mid-infrared human milk analyzer	—	HIGH (35–40°C; ultrasonic homogenizer (sonicator: 1.5 s/mL)	Mean ± SD: 3.1 ± 1.6 g/100 mL	2.5 ± 0.8 g/100 mL	−19.4%	0.0001
Total fatty acids	Ten-Doménech et al. [37]	UPLC	LOW (Gentle shaking)	—	Median: 230 (IQR = 120–290) mM	169 (104–252) mM	−26.5%	<0.01
SFA	Pitino et al. [25]	Folch extraction; GC	MODERATE (37°C; agitated and gently inverted to ensure homogeneity)	—	Mean ± SE: 1320 ± 104 mg/g	1260 ± 137 mg/g	Not significant	—
	Ten-Doménech et al. [37]	UPLC	LOW (Gentle shaking)	—	Median: 165 (IQR= 85–220) mM	123 (70–170) mM	−25.5%	<0.01
LCFA	Ten-Doménech et al. [37]	UPLC	LOW (Gentle shaking)	—	Median: 8.5 (IQR = 6.5–10) mM	7.2 (5.2–8.3) mM	−15.3%	<0.01
PUFA	Pitino et al. [25]	Folch extraction; GC	MODERATE (37°C; agitated and gently inverted to ensure homogeneity)	—	Mean ± SE: 558 ± 55 mg/g	536 ± 64 mg/g	Not significant	—
	Ten-Doménech et al. [37]	UPLC	LOW (Gentle shaking)	—	Median: 13 (IQR = 9–17) mM	11 (7.5 –15.5) mM	−15.4%	<0.01
MUFA	Pitino et al. [25]	Folsch extraction; GC	MODERATE (37°C; agitated and gently inverted to ensure homogeneity)	—	Mean ± SE: 1400 ± 150 mg/g	1310 ± 180 mg/g	Not significant	—
	Ten-Doménech et al. [37]	UPLC	LOW (Gentle shaking)	—	Median: 32 (IQR = 28–37) mM	28 (21–32) mM	−12.5%	<0.01
Specific fatty acids	Ten-Doménech et al. [37]	GC with quantification using external calibration	LOW (Gentle shaking)	—	Varied	Varied	Range: −38% to +17%	—

GC, gas chromatography; HoP, Holder pasteurization; LCFA, long-chain fatty acid; UPLC, ultraperformance liquid chromatography.

1 Time 1: Mixing conditions when drawing the baseline/raw sample.

2 Time 2: Mixing conditions when drawing the Holder pasteurized sample.

No mixing information was provided in 62% (16/26) of the studies (Table 3) [7,8,[14], [15], [16],^18^,^19^,^21^,^24^,[27], [28], [29], [30],^33^,^35^,^36^]. Total fat was assessed in all 16 studies, and 50% (8/16) observed a significant change of more than ±5% following pasteurization [7,8,19,27,29,33,35,36]. Interestingly, the degree of change ranged from −28.6% to +19.4%. One study also looked at fatty acids and reported no significant change [28].

**TABLE 3:** Summary by analyte for matched raw and Holder-pasteurized donor human milk among studies that did not report mixing information in the methods

Analyte	Author	Analysis method	Raw Value	HoP Value	% Change	*P*
Total fat	Escuder-Vieco et al. [30]	Fourier-transformed mid-infrared spectroscopy	34.7 (SEM:0.7) g/L	35.0 (0.5) g/L	Not significant	—
	Chang et al. [27]	Infrared analysis	Mean (SD): 3.1 ± 1.2 (g/dL)	2.5 ± 0.8 g/dL	−19.4%	<0.05
	Vincent et al. [28]	Mid-infrared human milk analyzer	Mean (SD): 23 ± 2.0 g/L	23 ± 2.0 g/L	Not significant	—
	Castro et al. [24]	Mid-infrared human milk analyzer	Mean: 3.1 g/dL	3.1 g/dL	Not significant	—
	Ley et al. [19]	Creamatocrit	Mean ± SD: 4.3 ± 0.95 g/L	3.9 ± 0.8 g/L	−9.3%	0.02
	Caballero Martín et al. [33]	Fourier-transformed mid-infrared spectroscopy	Mean (95% CI): 3.1 (3.0, 3.1) g/100 mL	2.9 (2.8−2.9)	−6.5%	0.00
	Vieira et al. [7]	Infrared analysis	Mean ± SD: 2.2 ± 1.5 mg%; median: 1.7	Mean: 2.1 ± 1.5 mg%; median: 1.7	−4.5%	<0.001
	Góes et al. [18]	Creamatocrit	Mean ± SD: 18.4 ± 13.2 g/L	18.6 ± 13.1 g/L	Not significant	—
	Martysiak-Żurowaska et al. [36]	Mid-infrared human milk analyzer	Mean ± SD: 3.6 ± 0.08 g/100 mL	3.8 ± 0.15 g/100 mL	5.6%	<0.05
	Lepri et al. [16]	Modified Folch Method	Mean ± SD: 24.7 ± 0.58 mg/mL	25.1 ± 0.5 mg/mL	1.6%	Not reported
	Williamson et al. [14]	Extraction (Van de Kamer)	Mean: 29.7 g/dL	30.9 g/dL	Not significant	—
	Jorge dos Santos et al. [35]	Folch method	Average total fat content in each lactation stage (g/100 g); colostrum: 2.0, transition: 3.1, mature: 4.5	Colostrum: 2.1, transition: 3.7, mature: 4.2	Colostrum: 5.0%, transition: 19.4%, mature: −6.7%	Not reported
	Friend et al. [15]	Roese-Gottlieb method	Mean: 2.80 g/100 mL	2.9 g/100 mL	Not significant	—
	Jorge dos Santos et al. [29]	Folch method	Mean ± SD (g/100 g): colostrum: 2.8 ± 0.81; transitional: 3.3 ± 0.8; mature: 3.5 ± 1.0	Colostrum: 2.4 ± 0.8; transitional: 2.9 ± 0.8; mature: 2.5 ± 1.2	Colostrum: −14.3%; transitional: −12.1%; mature: −28.6%	Not reported
	Kotrri et al. [21]	Near-infrared analysis	Pasteurized^1^ = 1.0 (raw) − 0.05 g/dL; *R*^2^ = 0.992	NA	Not significant	—
		Mojonnier extraction	Pasteurized^1^ = 0.98 (raw) ± 0.09 g/dL; *R*^2^ = 0.997	NA	Not significant	—
	Adhisivam et al. [8]	Mid-infrared human milk analyzer	Mean (SD): 3.6 ± 0.5 g/dL	2.7 ± 0.5 g/dL	−25.0%	0.001
Total fatty acids (both esterified and nonesterified)	Vincent et al. [28]	Chromatography coupled to flame ionization detector	Mean (SD): 22.8 ± 0.1 g/L	21.4 ± 0.8 g/L	Not significant	0.20

Abbreviations: NA, not available.

**FIGURE 3:**
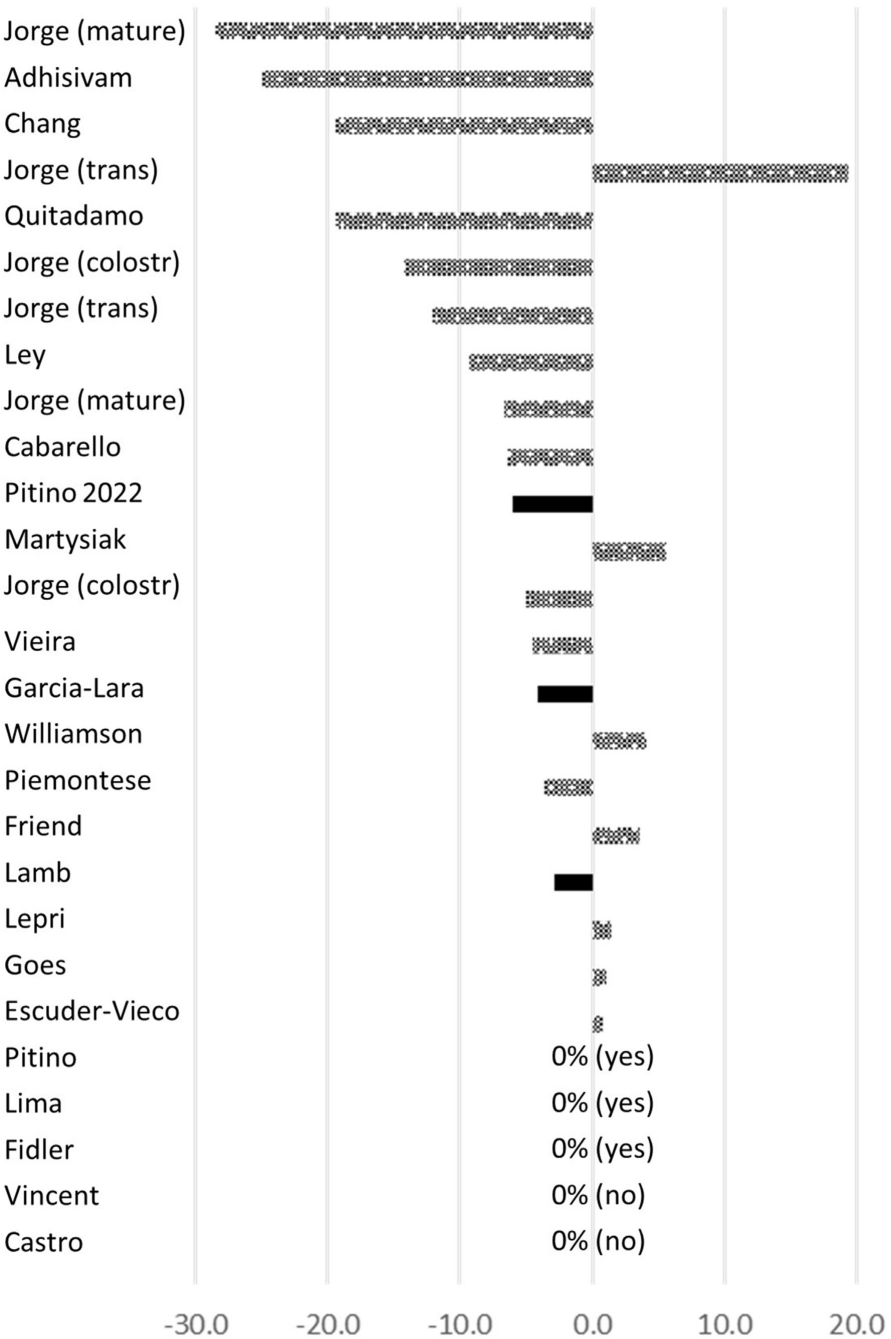
Range of percent fat change observed after Holder pasteurization by whether studies reported how the baseline (raw) sample was mixed (patterned bar = no; solid bar = yes). For studies where 0% change was observed, the mixing of raw samples is identified in parentheses.

Given the importance of beginning with a representative sample of raw milk, Figure 3 provides a visual summary of the observed changes reported in total fat after pasteurization, by whether the methods described mixing of the baseline/raw sample. The change in total fat reported when information was provided on raw sample mixing, independent of if these were deemed statistically significant, ranged from –6.0% to 0.0%, whereas the change reported when no mixing information was provided ranged from −28.4% to +19.4%.

## Discussion

Contrary to recent studies reporting a high loss of total fat after Holder pasteurization [8,27], our systematic scoping review of the peer-reviewed literature found that when study methodology described agitating DHM to combine the lipid and aqueous fractions, limited to no loss of total fat occurred following Holder pasteurization, providing important reassurances for clinicians using DHM with small, sick newborns. Data on the impact of Holder pasteurization on individual fatty acids and fatty acid classes were limited, with 1 low-quality-mixing study reporting a wide range of changes (−38% to +17%) [37]. It is important to note that the authors of this study reported accuracy in their methods ranging from 80% to 121% and precision from 1% to 20%; thus, some of the reported changes in individual fatty acids may have been driven by variability in the methods related to both analysis and mixing. Pitino et al. [25] quantified classes of fatty acids (e.g., saturated and monounsaturated) and reported no significant changes after pasteurization when using a moderate-quality mixing protocol. More research is needed on how Holder pasteurization impacts the quantity of fatty acids in DHM.

When mixing was described for raw milk, 17% (1/6) of studies saw a small change in total fat concentration following pasteurization (<5%). Alternatively, among studies that did not describe mixing at the first time point to collect a representative raw milk sample, 56% (10/18) reported a significant change >±5% of total fat concentration. Notably, although absence of information in the methods about mixing was not always associated with reported changes in fat after pasteurization, the studies that did not include mixing information consistently reported the greatest changes in total fat (Figure 3), with some studies reporting losses in excess of 25% and other studies reporting gains of nearly 20%. Conversely, when methods described mixing to ensure a representative sample of raw milk (Figure 3), fat loss was limited or did not occur. Thus, our review suggests that the incorporation of mixing methods with the intent of obtaining a representative sample may significantly influence the framing of the impact of Holder pasteurization.

Although the literature on the impact of mixing on nutrient distribution in human milk is limited, its impact on the behavior of lipids in milk samples has been explored by some. Fusch et al. [38] studied the impact of sample mixing (shaken by hand, vortexing, and ultrasonic homogenization) on the accuracy of infrared analysis. Infrared analysis quantifies macronutrients by transmitting light through a thin layer of liquid sample, thus milk fat must be evenly distributed to get accurate readings [39]. The report by Fusch et al. [38] found that mixing influenced the accuracy of fat measurements with infrared, with ultrasonication of samples producing more accurate results than handmixing and vortexing.

As discussed in the study by Fusch et al. [38], the impact of mixing on fat dispersion comes from the perspective of achieving specific conditions necessary for reliable and accurate infrared analysis—conditions that may not be necessary for other methods of fat analysis such as ether extraction, let alone the collection of a representative sample. Further, although ultrasonic homogenization is represented as a mixing method among studies in this scoping review, it may not speak to the type of mixing that occurs in milk banks where it is more common to mix pools by hand or by mechanically stirring to minimize potential damage to bioactive substances [40].

The analysis of mixing techniques often observed in human milk banks is a focal point in the study by Friend and Perrin [11], who aimed to assess the distribution of total fat and other components of DHM samples when large volumes of milk were mixed with either manual (hand swirling) or mechanical (magnetic stir plate) techniques compared with an unmixed (control) group. Total fat concentration among samples drawn from manually and mechanically mixed pools differed from their respective pool means by at most 5%, which was significantly lower than the control group samples, which differed by ≤20%. This suggests that the quality of mixing may not be as predictive of fat loss as would a situation that does not include any mixing, which is in agreement with the findings of this review.

Additionally, research conducted by the dairy industry supports the notion that undisturbed milk separates into skim and fat factions, which can impact the distribution of fat during bottling [41]. Both Fusch et al. [38] and Friend and Perrin [11] consider that a delay, or hold period between mixing and drawing a sample or conducting analysis may contribute to variations in human milk fat. For instance, Fusch et al. [38] observed that fat molecules were reaggregating within minutes when using handmixing and vortexing, contributing to inaccuracies of 0.5 g/dL or higher in fat readings using infrared analysis. Friend and Perrin [11] noted that the total fat concentrations were more variable after DHM pools were held at 4 °C for 24 h before bottling, than pools that were bottled within 1 h, despite using the same degree of manual mixing. Thus, collecting a representative sample of human milk during research studies is likely a function of both sample mixing and hold-time.

DHM processing is multifactorial, and several steps can potentially impact the fat content between samples. Inconsistencies in research methodology and sample handling, reflected in the studies selected for this review, likely contribute to the disparate findings on the impact of Holder pasteurization on the composition of lipid-based constituents in DHM. This review suggests that the absence of mixing protocols may correlate with an increased occurrence of a change in total fat concentration following Holder pasteurization (Figure 3) compared with when mixing of any quality is applied. Interestingly, this analysis suggests that the time at which mixing is used (before drawing a representative raw or Holder-pasteurized sample) is a strong predictor of whether studies will report null compared with significant findings, highlighting the methodologic importance of mixing when studying lipids in human milk. A limitation of this review is that absence of methodologic details on mixing does not necessarily mean mixing did not occur. Future studies should explicitly state the mixing protocols to help put findings in context.

Our systematic review of the peer-reviewed literature found that there was limited or no loss of total fat in DHM following Holder pasteurization among studies that described methods for agitating the sample to combine the lipid and aqueous fractions that naturally separate in DHM. Future research is needed to clarify the impact of mixing protocols throughout the process of producing DHM (larger volumes of DHM mixed in a milk bank) and using DHM (smaller volumes of DHM mixed in a clinical setting), which will ultimately help to improve preterm infant feeding. More research is also warranted on specific lipids within the lipidome, given that previous Holder pasteurization research has focused primarily on total fat.

## Author contributions

The authors’ responsibilities were as follows – ARD, MTP: contributed to the conception of the research; ARD: conducted the search; ARD, MTP: reviewed abstracts and manuscripts, abstracted the data, and interpreted the findings; ARD: drafted the manuscript; MTP: edited the manuscript; and both authors read and approved the final manuscript.

## Conflict of interest

Both authors report no conflicts of interest.

## Funding

The authors reported no funding received for this study.

## References

[1] Meek J.Y., Noble L. (2022). Section on Breastfeeding, Policy statement: breastfeeding and the use of human milk. Pediatrics.

[2] PATH (2013). Strengthening human milk banking: a global implementation framework.

[3] Committee on Nutrition (2017). Section on Breastfeeding, Committee on Fetus and Newborn, Donor human milk for the high-risk infant: preparation, safety, and usage options in the United States. Pediatrics.

[4] Updegrove K., Festival J., Hackney R., Jones F., Kelly S., Sakamoto P. (2020). HMBANA standards for donor milk banking: an overview.

[5] Quigley M., Embleton N.D., McGuire W. (2019). Formula versus donor breast milk for feeding preterm or low birth weight infants. Cochrane Database Syst Rev.

[6] Peila C., Moro G.E., Bertino E., Cavallarin L., Giribaldi M., Giuliani F. (2016). The effect of Holder pasteurization on nutrients and biologically-active components in donor human milk: a review. Nutrients.

[7] Vieira A.A., Soares F.V.M., Pimenta H.P., Abranches A.D., Moreira M.E.L. (2011). Analysis of the influence of pasteurization, freezing/thawing, and offer processes on human milk’s macronutrient concentrations. Early Hum. Dev..

[8] Adhisivam B., Vishnu Bhat B., Rao K., Kingsley S.M., Plakkal N., Palanivel C. (2019). Effect of Holder pasteurization on macronutrients and immunoglobulin profile of pooled donor human milk. J. Matern. Fetal Neonatal Med..

[9] Parat S., Raza P., Kamleh M., Super D., Groh-Wargo S. (2020). Targeted breast milk fortification for very low birth weight (VLBW) infants: nutritional intake, growth outcome and body composition. Nutrients.

[10] (2019). Pediatric Nutrition Dietetics Practice Group, Infant and pediatric feedings: guidelines for preparation of human milk and formula in health care facilities.

[11] Friend L.L., Perrin M.T. (2021). Methods of mixing donor human milk during bottling results in fat differences between samples within a pool. J. Dairy Sci..

[12] Arksey H., O’Malley L. (2005). Scoping studies: towards a methodological framework. Int. J. Soc. Res. Methodol..

[13] USDA, Commercial Items Description (CID). Milks, fluid [Internet]. [cited 2024 Mar 24]. Available from: https://www.ams.usda.gov/sites/default/files/media/CID%20Milks%2C%20Fluid.pdf.

[14] Williamson S., Finucane E., Ellis H., Gamsu H.R. (1978). Effect of heat treatment of human milk on absorption of nitrogen, fat, sodium, calcium, and phosphorus by preterm infants. Arch. Dis. Child.

[15] Friend B.A., Shahani K.M., Long C.A., Agel E.N. (1983). Evaluation of freeze-drying, pasteurization, high-temperature heating and storage on selected enzymes, B-vitamins and lipids of mature human milk. J. Food Prot..

[16] Lepri L., Del Bubba M., Maggini R., Donzelli G.P., Galvan P. (1997). Effect of pasteurization and storage on some components of pooled human milk. J. Chromatogr. B. Biomed. Sci. App..

[17] Fidler N., Sauerwald T.U., Demmelmair H., Koletzko B. (2001). Fat content and fatty acid composition of fresh, pasteurized, or sterilized human milk. Adv. Exp. Med. Biol..

[18] Góes H.C.A., Torres A.G., Donangelo C.M., Trugo N.M.F. (2002). Nutrient composition of banked human milk in Brazil and influence of processing on zinc distribution in milk fractions. Nutrition.

[19] Ley S.H., Hanley A.J., Stone D., O’Connor D.L. (2011). Effects of pasteurization on adiponectin and insulin concentrations in donor human milk. Pediatr. Res..

[20] García-Lara N.R., Vieco D.E., De la Cruz-Bértolo J., Lora-Pablos D., Velasco N.U., Pallás-Alonso C.R. (2013). Effect of Holder pasteurization and frozen storage on macronutrients and energy content of breast milk. J. Pediatr. Gastroenterol. Nutr..

[21] Kotrri G., Fusch G., Kwan C., Choi D., Choi A., Al Kafi N. (2016). Validation of correction algorithms for near-IR analysis of human milk in an independent sample set—effect of pasteurization. Nutrients.

[22] Lima H.K., Wagner-Gillespie M., Perrin M.T., Fogleman A.D. (2017). Bacteria and bioactivity in holder pasteurized and shelf-stable human milk products. Curr. Dev. Nutr..

[23] Piemontese P., Mallardi D., Liotto N., Tabasso C., Menis C., Perrone M. (2019). Macronutrient content of pooled donor human milk before and after Holder pasteurization. BMC Pediatr.

[24] Castro M., Asbury M., Shama S., Stone D., Yoon E.W., O’Connor D.L. (2019). Energy and fat intake for preterm infants fed donor milk is significantly impacted by enteral feeding method. JPEN J. Parenter. Enteral. Nutr..

[25] Pitino M.A., Alashmali S.M., Hopperton K.E., Unger S., Pouliot Y., Doyen A. (2019). Oxylipin concentration, but not fatty acid composition, is altered in human donor milk pasteurised using both thermal and non-thermal techniques. Br. J. Nutr..

[26] Pitino M.A., Unger S., Doyen A., Pouliot Y., Aufreiter S., Stone D. (2019). High hydrostatic pressure processing better preserves the nutrient and bioactive compound composition of human donor milk. J. Nutr..

[27] Chang F.Y., Fang L.J., Chang C.S., Wu T.Z. (2020). The effect of processing donor milk on its nutrient and energy content. Breastfeed Med.

[28] Vincent M., Ménard O., Etienne J., Ossemond J., Durand A., Buffin R. (2020). Human milk pasteurisation reduces pre-lipolysis but not digestive lipolysis and moderately decreases intestinal lipid uptake in a combination of preterm infant in vitro models. Food Chem.

[29] Jorge dos Santos V., Baqueta M.R., Neia V.J.C., Magalhães de Souza P., Março P.H., Valderrama P. (2021). MicroNIR spectroscopy and multivariate calibration in the proximal composition determination of human milk. LWT.

[30] Escuder-Vieco D., Rodríguez J.M., Espinosa-Martos I., Corzo N., Montilla A., García-Serrano A. (2021). High-temperature short-time and holder pasteurization of donor milk: impact on milk composition. Life (Basel).

[31] Lamb R.L., Haszard J.J., Little H.M.J., Franks A.F., Meeks M.G. (2021). Macronutrient composition of donated human milk in a New Zealand population. J. Hum. Lact.

[32] Quitadamo P.A., Sorrentino L., Palumbo G., Cianti L., Copetti M., Gentile M.A. (2021). Effect of Holder pasteurization on macronutrients and energy content of pooled donor human milk. J. Pediatr. Neonatal Individ. Med..

[33] Caballero Martín S., Del Sánchez Gómez de Orgaz M.D.C., Sánchez Luna M. (2022). Quality study of Holder pasteurization of donor human milk in a neonatal personalized nutrition unit. Ann. Paediatr. (Engl ed)..

[34] Pitino M.A., Unger S., Gill A., McGeer A.J., Doyen A., Pouliot Y. (2022). High pressure processing inactivates human cytomegalovirus and hepatitis A virus while preserving macronutrients and native lactoferrin in human milk. Innov. Food Sci. Emerg. Technol..

[35] Jorge dos Santos V., Baqueta M.R., Março P.H., Valderrama P., Visentainer J.V. (2022). Proof-of-concept on the effect of human milk storage time: lipid degradation and spectroscopic characterization using portable near-infrared spectrometer and chemometrics. Food. Chem..

[36] Martysiak-Żurowska D., Malinowska-Pańczyk E., Orzołek M., Kusznierewicz B., Kiełbratowska B. (2022). Effect of microwave and convection heating on selected nutrients of human milk. Food Chem..

[37] Ten-Doménech I., Ramos-Garcia V., Moreno-Torres M., Parra-Llorca A., Gormaz M., Vento M. (2022). The effect of Holder pasteurization on the lipid and metabolite composition of human milk. Food Chem..

[38] Fusch G., Rochow N., Choi A., Fusch S., Poeschl S., Ubah A.O. (2015). Rapid measurement of macronutrients in breast milk: how reliable are infrared milk analyzers?. Clin. Nutr..

[39] MIRIS Human Milk Analyzer© User Manual [Internet]. [cited 2023 Aug 2]. Available from: http://www.mirissolutions.com/support/user-manuals.

[40] Friend L.L., Perrin M.T. (2020). Fat and protein variability in donor human milk and associations with milk banking processes. Breastfeed Med.

[41] Walstra P. (1999).

